# One-pot synthesis of iron oxide - Gamma irradiated chitosan modified SBA-15 mesoporous silica for effective methylene blue dye removal

**DOI:** 10.1016/j.heliyon.2023.e16178

**Published:** 2023-05-10

**Authors:** Titiya Meechai, Thinnaphat Poonsawat, Nunticha Limchoowong, Sakchai Laksee, Peerapong Chumkaeo, Ranida Tuanudom, Artitaya Yatsomboon, Lalita Honghernsthit, Ekasith Somsook, Phitchan Sricharoen

**Affiliations:** a Department of Premedical Science, Faculty of Medicine, Bangkokthonburi University, Thawi Watthana, Bangkok 10170, Thailand; b NANOCAST Laboratory, Center for Catalysis Science and Technology (CAST), Department of Chemistry and Center of Excellence for Innovation in Chemistry, Faculty of Science, Mahidol University, 272 Rama VI Rd., Ratchathewi, Bangkok 10400, Thailand; c Department of Chemistry, Faculty of Science, Srinakharinwirot University, Bangkok 10110, Thailand; d Nuclear Technology Research and Development Center, Thailand Institute of Nuclear Technology (Public Organization), Nakhon Nayok 26120, Thailand

**Keywords:** SBA-15, Gamma irradiated chitosan, Methylene blue dye, Mesoporous silica

## Abstract

The development of adsorption technology and the processing of radiation have both been influenced by chitosan adsorbent (γ-chitosan), a raw material with unique features. The goal of the current work was to improve the synthesis of Fe-SBA-15 utilizing chitosan that has undergone gamma radiation (Fe-γ–CS–SBA-15) in order to investigate the removal of methylene blue dye in a single hydrothermal procedure. High-resolution transmission electron microscopy (HRTEM), High angle annular dark field scanning transmission electron microscopy (HAADF-STEM), small- and wide-angle X-ray powder diffraction (XRD), Fourier transform-infrared spectroscopy (FT-IR) and Energydispersive X-ray spectroscopy (EDS) were used to characterize γ–CS–SBA-15 that had been exposed to Fe. By using N_2_-physisorption (BET, BJH), the structure of Fe-γ–CS–SBA-15 was investigated. The study parameters also included the effect of solution pH, adsorbent dose and contact time on the methylene blue adsorption. The elimination efficiency of the methylene blue dye was compiled using a UV-VIS spectrophotometer. The results of the characterization show that the Fe-γ–CS–SBA-15 has a substantial pore volume of 504 m^2^ g^−1^ and a surface area of 0.88 cm^3^ g^−1^. Furthermore, the maximum adsorption capacity (Q_max_) of the methylene blue is 176.70 mg/g. The γ-CS can make SBA-15 operate better. It proves that the distribution of Fe and chitosan (the C and N components) in SBA-15 channels is uniform.

## Highlights

• One - Pot synthesis of iron oxide - gamma irradiated chitosan modified SBA-15.
• Fe-γ–CS–SBA-15 adsorbent dose used to better bind methylene blue than Fe-SBA-15.
• Chitosan that has undergone gamma radiation is used to boost SBA-15's potential.
• Fe-γ–CS–SBA-15 shown honeycomb-style network of mesoporous materials.

## 1 Introduction

Wastewater from the dyeing process is present in the majority of dyeing plants. Remaining paint and chemical pollutants therefore have an impact on water pollution issues. Typically, the complicated compounds with a big complex formula and possibly harmful materials utilized in fabric dyes used in dyeing facilities. Color may be removed from dyeing effluents using a variety of techniques, including biological degradation, chemical precipitation, ozone usage, and adsorption [[1], [2], [3]]. Another extremely effective strategy involves employing a regenerable heterogeneous catalyst and iron ions in the form of Fe^2+^ or Fe^3+^ to create compounds as a catalyst in the Fenton reaction [[4], [5], [6]]. Due to their highly structured porosity and large surface area, which is employed as an adsorbent, MCM-41 and SBA-15 [[7], [8], [9], [10], [11], [12], [13], [14]] are the two types of silica mesopores that are most often manufactured. Fe-SBA15 was synthesized in order to enhance the adsorption of antibiotic tetracycline by Zhang et al. [15] and the use of Fe-MCM-41 for production of carbon nanotubes was studied by Amama et al. [16]. The recyclability of these catalysts and the fact that mesoporous silica's pore diameters mostly rely on the structural component are two of its advantages. The majority of structure-directing substances are pricy synthetic compounds with significant environmental effect. As a result, several studies have been done to develop substitute materials, with chitosan [[17], [18], [19], [20], [21]] being one among them. Chitosan is a naturally occurring biopolymer that is found in the exoskeletons of animals including shrimp, crabs, insects, and fungus [22]. It is a chemical that may naturally biodegrade. Chitosan that has undergone irradiation has had its molecules lightened by gamma radiation, which causes the breaking of chemical bonds. Because of the smaller chitosan molecules, plants can absorb and use it more quickly than they can with regular chitosan [23,24]. The radiation utilized quickly dissipates, rendering it harmless to people, pets, and the environment. Applications involve chitosan with a lower molecular weight but a chemically intact structure. Under the influence of an electron or γ-ray beam, chitosan breaks down into little pieces. Low molecular weight chitosan has reportedly been found to have significantly greater solubility, growth-promoting characteristics, and electrical properties than high molecular weight chitosan [25]. γ-Irradiated chitosan is used by Mirajkar et al. to deploy γ-irradiated chitosan–silver nanocomposites for the control of phytopathogens and to research the antibacterial activity of IR–CSN–Ag NPs [24]. The interaction of the silanol groups (SiOH) and organic groups NH_2_ of chitosan produces the skeleton compounds with functional decorating for the inner and wall of mesoporous silica to increase its particular performance. The hydroxyl groups on the surface of silica mesopores (silicon oxide). The chitosan restricts the silica, forming a lattice that forces it to expand its internal network and produce many holes. Therefore, it may be used for a variety of purposes [26]. Mesoporous silica-chitosan composites were prepared by Cui et al. [27] using a one-pot microwave assisted process to serve as an adsorbent for the recovery of Re(VII) in actual industrial effluents. Additionally, Machado et al. [28] used the sol-gel method to produce silica/chitosan-based composites and to test the effects of crosslinking chitosan with glutaraldehyde. This study used silica mesopore type SBA-15 modified with iron oxide and gamma-irradiated chitosan to remove methylene blue dye. The Fe-γ–CS–SBA-15 was analyzed by High-resolution transmission electron microscopy (HRTEM), High angle annular dark field scanning transmission electron microscopy (HAADF-STEM), small- and wide-angle X-ray powder diffraction (XRD), Energy dispersive X-ray spectroscopy (EDS), Fourier transform-infrared spectroscopy (FT-IR) and N_2_-physisorption (BET, BJH). The criteria of the investigation were the impact of adsorbent dose, contact time and solution pH. Using a UV-VIS Spectrophotometer to measure the concentration of the methylene blue dye.

## 2 Experimental

### 2.1 Materials and reagents

Sigma-Aldrich Co., USA, provided the nonionic surfactant of EO20PO70EO20, (Pluronic P123), ethanol (99%), TEOS (tetraethyl orthosilicate), FeCl_3_, and hydrochloric acid (HCl, 37%). The majority (>96%) of MB's supplies came from Central Drug House (P) Ltd. In India. The supplier of chitosan was Biolife in Thailand.

### 2.2 Apparatus and instruments

Powder X-ray diffraction patterns were achieved on a Bruker D8 Advance A25 diffractometer equipped with a Ni filter (Cu K radiation: 0.154184 nm) and a one-dimensional multistrip detector (Lynxeye, 192 channels on 2.95°). Transmission electron microscopy (HRTEM) was performed using a JEOL JEM-ARM200F microscope equipped with a high-angle annular dark-field (HAADF) detector and an energy-dispersive spectrometer (EDS). Brunauer-Emmett-Teller (BET) surface area was measured with N_2_ as adsorbate at 77.3 K by Quantachrome Instruments v11.0. Fourier transform-infrared spectroscopy (FT-IR) was performed on Bruker Hong Kong Limited model ALPHA. The Agilent 7500ce (USA) inductively coupled plasma-mass spectrometry (ICP-MS) was used to analysis of Fe element [29].

### 2.3 Synthesis of Fe-γ- irradiated *C*S-SBA-15

Pluronic 123 was used as the structure-directing agent during the synthesis of Fe-γ–CS–SBA-15 in an acidic environment [30,31]. To create the pluronic 123 solution, 4 g of pluronic 123 were dissolved in 60 g of HCl (4 M), then the mixture was stirred at 40 °C until the pluronic 123 was completely dissolved [[32], [33], [34]]. TEOS was gradually added and mixed for 40 min at 40 °C. Then, the mixtures between 50 mL of 0.1 M FeCl_3_ (Si 35 mmol: Fe 5 mmol) [35,36] and 0.5 g of γ-CS was added slowly and stirred at 40 °C for 20 h. The gel mixture was put into a Teflon container and matured for 24 h at 100 °C [[37], [38], [39], [40]]. The solid was filtered out and repeatedly washed with an ethanol mixture after separation. The solid sample was then dried for 12 h at 80 °C under vacuum. Soxhlet extraction with ethanol for 48 h and drying in an oven at 100 °C were used to remove the surfactant [[41], [42], [43], [44]].

### 2.4 Adsorption experiments

50 mL of 100 mg/L MB solution was first made, and 50 mg of Fe-γ–CS–SBA-15 was subsequently added. After stirring the combination, the amount of adsorption was determined by sampling and filtering the solution. Samples' absorbance was determined using an *S*P-UV200 UV/VIS Spectrophotometer at 664 nm. A universal pH buffer for the pH range of 2–12, was used to change the pH of solutions [[45], [46], [47]]. Using a formula that calculates the % removal effectiveness of methylene blue, the adsorption of the samples was examined.

(1) Removal efficiency = [(C_0_–C)/ C_0_] × 100

Where C_0_ is the initial concentration of methylene blue, C is the solution concentration after adsorption at any time [[48], [49], [50]].

## 3 Results and discussion

### 3.1 Characterization of Fe-γ–CS–SBA-15

**Fig. 1:**
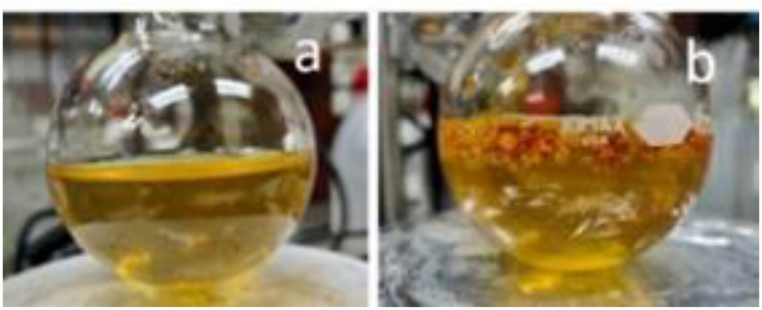
Solubility of (a) gamma irradiated chitosan and (b) non-irradiated chitosan.

**Fig. 2:**
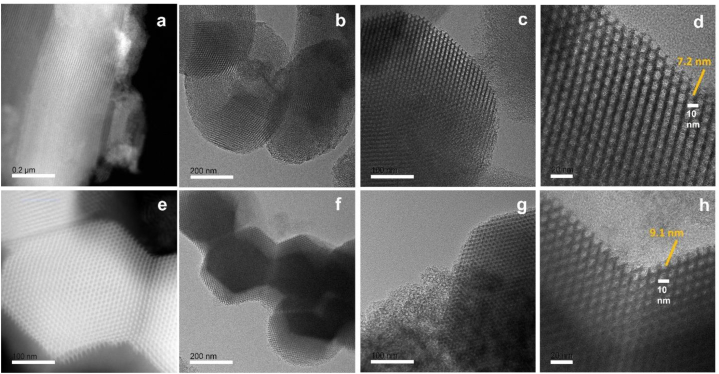
HRTEM and HAADF-STEM images: (a) HAADF-STEM image and (b)–(c) HRTEM image of Fe-SBA-15; (e) HAADF-STEM image and 2(f)–(h) HRTEM image of Fe-γ–CS–SBA-15.

**Fig. 3:**
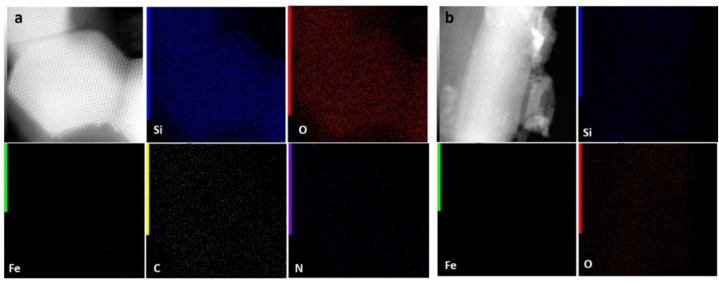
EDS mapping of Fe-γ–CS–SBA-15 (a) and Fe-SBA-15 (b).

**Fig. 4:**
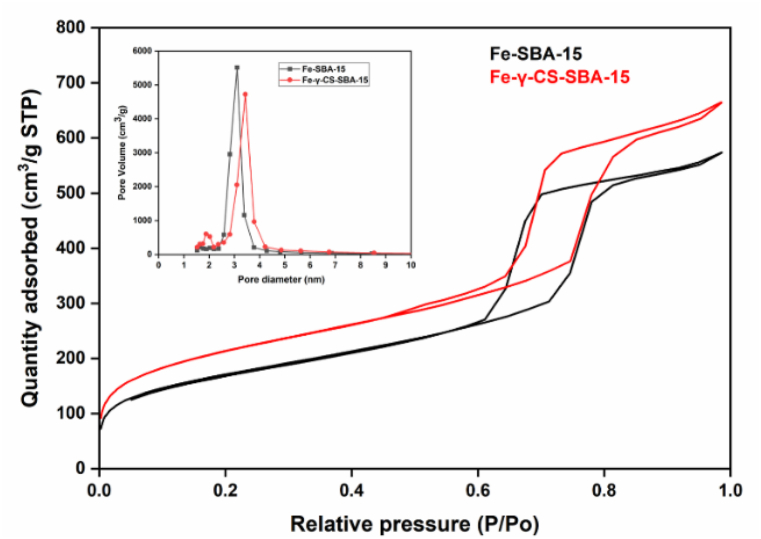
The nitrogen adsorption–desorption isotherms of Fe-SBA-15 and Fe-γ–CS–SBA-15.

**Fig. 5:**
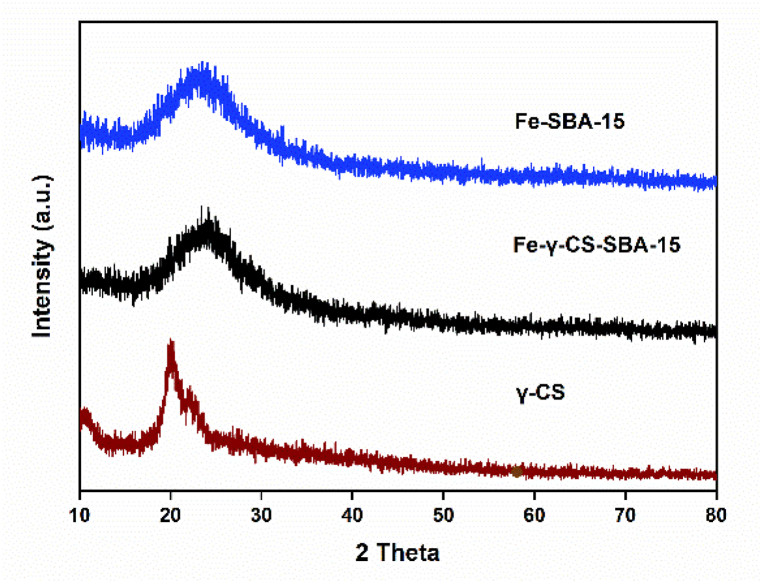
The small angle XRD patterns of Fe-SBA-15 and Fe-γ–CS–SBA-15.

**Fig. 6:**
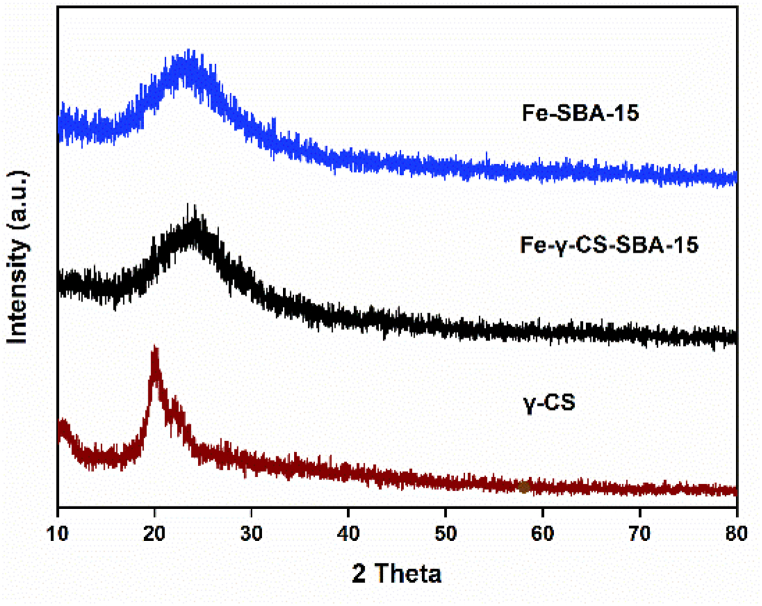
The wide angle XRD patterns of Fe-SBA-15, Fe-γ–CS–SBA-15 and γ-irradiated chitosan.

**Fig. 7:**
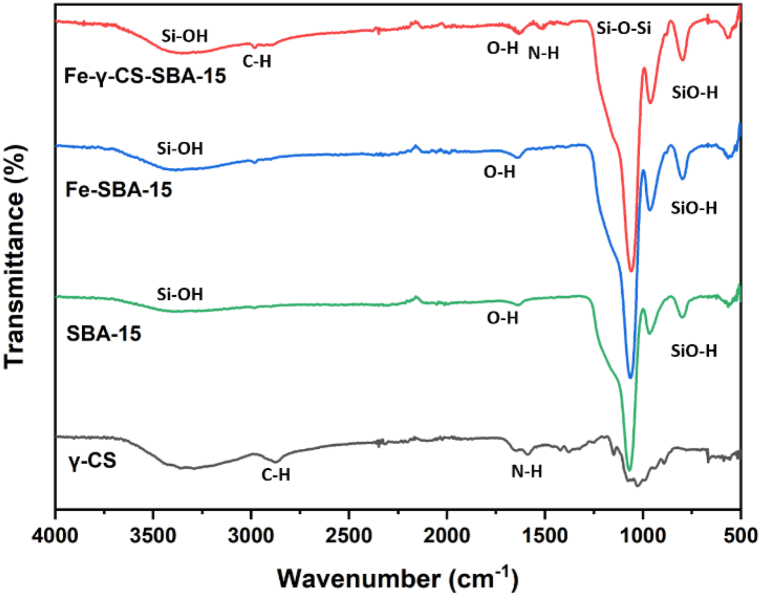
FTIR spectra of γ-CS, SBA-15, Fe-SBA-15 and Fe-γ–CS–SBA-15.

**Fig. 8:**
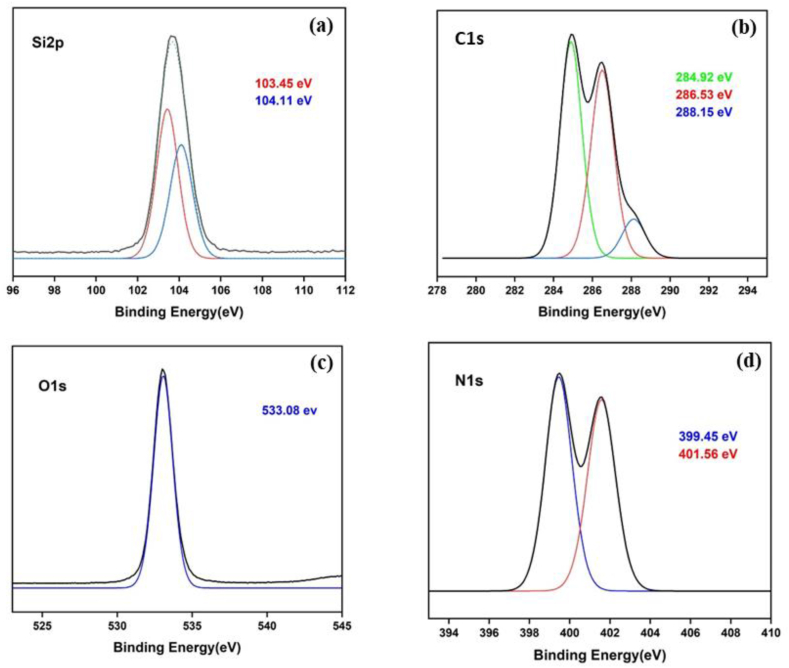
XPS spectrum of Fe-γ–CS–SBA-15. (a) Si2p, (b) C1s, (c) O1s and (d) N1s.

**Fig. 9:**
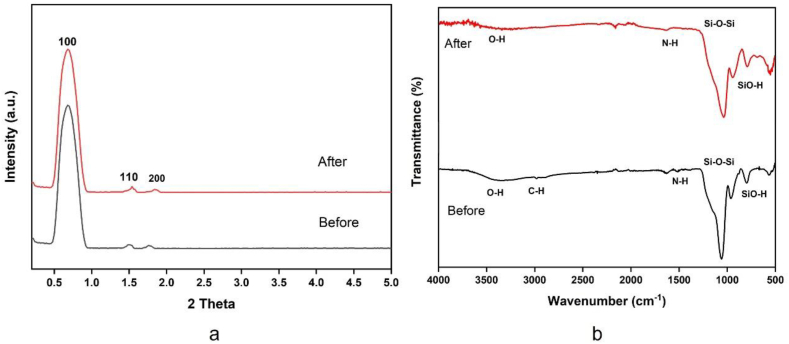
Before and after used Fe-γ–CS–SBA-15. (a) XRD patterns and (b) FTIR spectra.

Typically, SBA-15 is created via the SBA-15 synthesis in an acidic environment. Therefore, chemicals that ought to be highly soluble in acid are utilized to improve their structure or other attributes [51,52]. Fig. 1 illustrates the findings, which demonstrated that gamma-irradiated chitosan (Fig. 1(a)) was more soluble than non-irradiated chitosan (Fig. 1(b)) in an acidic environment. In this study, chitosan that has undergone gamma radiation is used to boost SBA-15's potential. The HRTEM and HAADF-STEM images were displayed in Fig. 2(a–h). It supports the honeycomb-shaped network of SBA-15 [[53], [54], [55]]. Pore sizes of 7.2 and 9.1 nm for the mesoporous silicas Fe-SBA-15 and Fe-γ–CS–SBA-15, respectively, were estimated from numerous HRTEM images. Fe, Si, O, C, and N elements were identified using EDS mapping of Fe-γ–CS–SBA-15 (Fig. 3(a)) and Fe-SBA-15 (Fig. 3(b)). The EDS results showed that gamma-irradiated chitosan synthesis had a greater percentage of Fe (3.90%) than that of chitosan-free synthesis (Fe 2.27%), which may mean that chitosan may contribute more Fe to the structure (Table 1). Additionally, it shows how evenly Fe and chitosan (the C and N components) are dispersed within SBA-15 channels. Fig. 4 depicts the N_2_ adsorption/desorption isotherms for Fe-SBA-15 and Fe-γ–CS–SBA-15 together with the BJH pore size distributions for each material. Fig. 4 further demonstrates that the average pore size increased even though the dispersion remained almost the same. Compared to Fe-SBA-15, Fe-γ–CS–SBA-15 has a wider pore size dispersion. In order to improve pore diameter, it was demonstrated that direct synthesis required the direct insertion of a small amount of gamma-irradiated chitosan into the skeletal structure of SBA-15 [56]. The HRTEM figure's pore diameters (7.2 and 9.1 nm) were larger than the BJH techniques' calculations of BET (3.1 and 3.4 nm), however it can still be said that the gamma chitosan addition expanded the pore structure. According to IUPAC categorization, the sample displays a type IV isotherm and a type-H1 hysteresis loop feature. The findings of the characterization demonstrate that Fe-γ–CS–SBA-15 has a large pore volume, 504 m^2^/g, and 0.88 cm^3^/g (Table 2). A significant diffraction peak at 2θ of 0.68° is shown in small angle XRD patterns (Fig. 5). This peak may be indexed using a hexagonal array of mesopores similar to SBA-15 [55,57]. Moreover, the d_100_ interplanar spacings can be calculated to be 12.98 that Fe-SBA-15 and Fe-γ–CS–SBA-15 are the same d_100_ [58]. Both reflections yield a unit cell parameter of a_0_ = 14.98 nm, where a_0_ represents the pore-to-pore distance of the hexagonal structure. It suggests that the organized mesoporous structure is not destroyed by the addition of gamma-irradiated chitosan. Fig. 6 displays the wide angle XRD patterns of Fe-SBA-15 and Fe-γ–CS–SBA-15, which are typical of mesoporous SBA-15 and have just one broad peak between 2θ values of 20–30° [58]. However, they disappeared the γ-CS and Fe diffraction peaks. Materials with iron substitution in SBA-15 (Fe-SBA-15) have been synthesized using a straightforward direct hydrothermal process and minimally acidic conditions. It could be due to SBA-15, which contains Fe, having low Fe loading, which causes it to surpass the XRD detection threshold. Fig. 7 displays the FTIR spectra of γ-CS, SBA-15, Fe-SBA-15 and Fe-γ–CS–SBA-15, which have significant intensity peaks at 798 cm^−1^ due to the presence of SiO–H groups. The silanol groups Si–*O*–Si bands are attributed to the peaks at 1062 and 557 cm^−1^. Due to the bending mode of OH, including water H–O–H, the peak at 1632 cm^−1^ is caused. These functional groups match the FTIR pattern of SBA-15 from TEOS [59,60]. An additional band at 1541 cm^−1^, corresponding to N–H bending, was found in the spectra of Fe-γ–CS–SBA-15; however, this band is also used to describe the C–N of chitosan [61]. Fig. 8(a–d) depicts the Fe-γ–CS–SBA-15 XPS spectrum. The lattice oxygen of silica is responsible for the first peak with a higher binding energy of 533.0 eV. A prominent signal at 103.5 eV, which is typical of SiO_2_-based materials, can be seen in the Si2p spectrum. Three elements in the C1s spectrum have chemical shifts that correspond to the following groups: aliphatic (C–C, C–H) groups (284.9 eV (0.1)), CH and C=N groups (286.5 eV (±0.1)), and C=O groups (288.1 eV (±0.1)) [62,63]. The amino groups that were engaged in hydrogen bonds (NH_2_–O) were assigned the binding energies of the N 1 s band at 399.5 eV [64]. Additionally, it was discovered that this band's binding energies of 401.56 eV were associated with chelation between amino groups and iron ions (NH_2_–Fe). The XPS findings agree with the structural data for SBA-15 and chitosan [65]. Due to the strong dispersion in the extremely porous material surfaces, it was not possible to determine the binding energy of Fe2p for XPS measurements; as a result, it is challenging to identify Fe on the material surface by XPS. Fe may be detected with clarity in ICP-MS experiments to confirm its existence. the Fe contents of several materials were determined. Fe-SBA-15 and Fe-γ–CS–SBA-15 had Fe loading efficiencies of 11.28 mg/kg and 21.68 mg/kg, respectively. According to the EDX and ICP data, which indicated that iron was present in the samples, the addition of γ-CS improved the iron's ability to attach to the structure and increase the potential. The results of the small angle XRD patterns (Fig. 9(a)) and FTIR spectra before and after using Fe-γ–CS–SBA-15 show that the structure of the compound was not changed (Fig. 9(b)).

**Table 1:** EDS analysis of Fe-SBA-15 and Fe-γ–CS–SBA-15.

Catalysts	Atomic %				
	Si	O	Fe	C	N
Fe-SBA-15	39.57	58.16	2.27	–	–
Fe-γ–CS–SBA-15	28.02	50.14	3.90	17.04	0.90

**Table 2:** Textural properties of the catalysts (BET and BJH methods). Notes: ^a^BET surface area and ^b^BJH methods.

Samples	Surface Area^a^, S_BET_ (m^2^/g)	Pore Volume^b^, Vp (cm^3^/g)	Average Pore diameter^b^, Dp (nm)
Fe-SBA-15	487	0.79	3.1
Fe-γ–CS–SBA-15	504	0.88	3.4

### 3.2 Adsorption

**Fig. 10:**
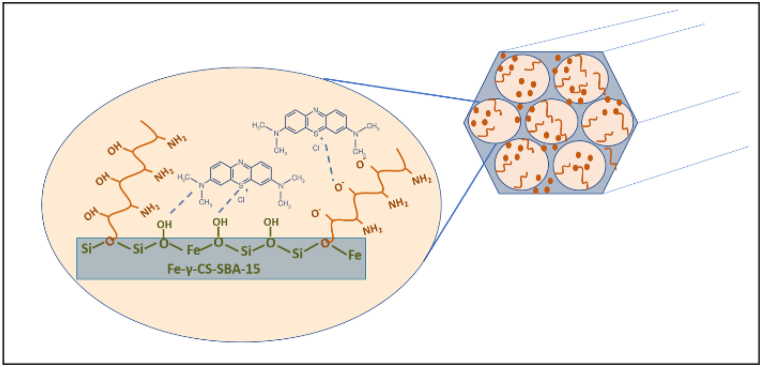
Proposed interaction scheme between Fe-γ–CS–SBA-15 and MB dye.

**Fig. 11:**
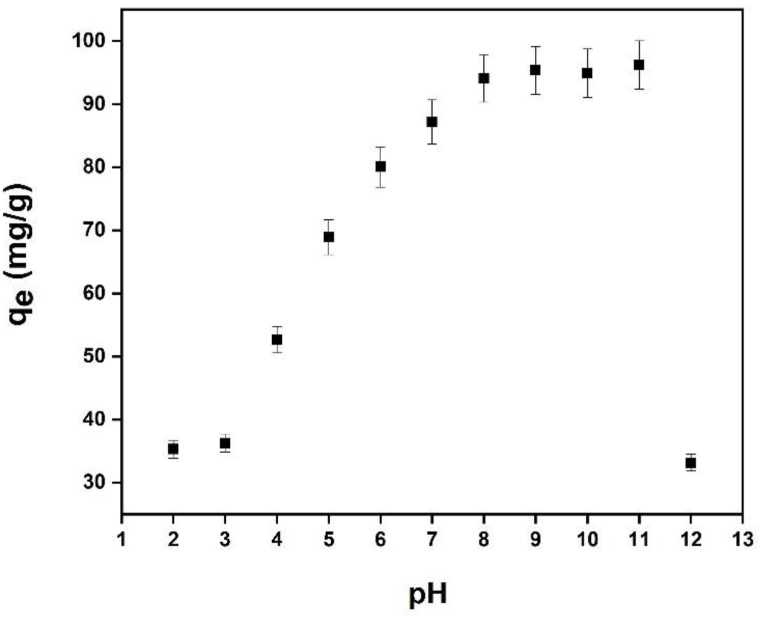
Effect of solution pH value on the adsorption of methylene blue, (Fe-γ–CS–SBA-15 0.05 g, [MB] 100 mg/L, Time 1 h).

**Fig. 12:**
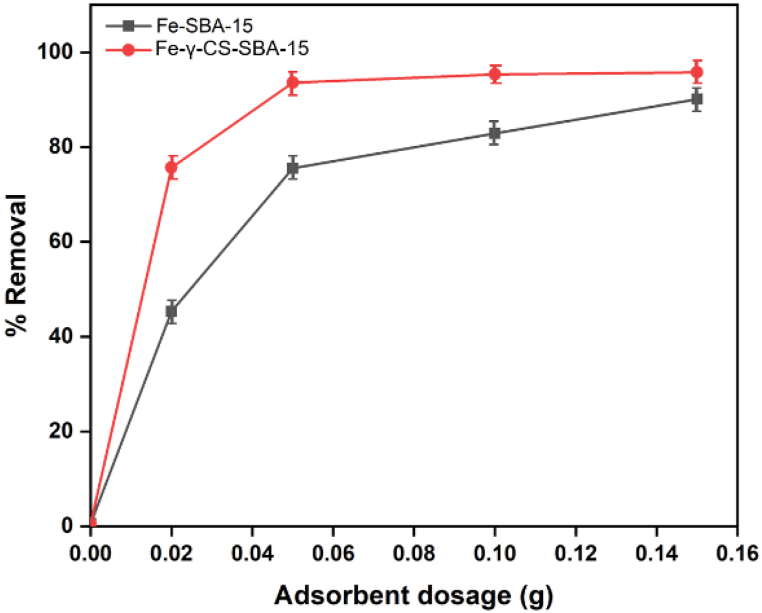
Effect of adsorbent dosage on adsorption of methylene blue ([MB] 100 mg/L, pH 9, Time 30 min)

The capacity of the sample to adsorb MB was discovered to increase when the original pH increased. The lower absorption at low pH is caused by the large concentration of H^+^ ions in the solution, which compete with methylene blue, which is also positively charged. Thus, when the methylene blue dye was removed, pH 9 was used to investigate further factors. The SBA-15 surface –OH lost H^+^ due to the alkaline state, and a significant quantity of negative charge was present on the material. And because methylene blue is a basic cationic dye that ionizes to produce colored cations in aqueous solution, SBA-15, which has a lot of negative charges, is coupled with the methylene blue-ionized colored cations to produce the adsorption effect. In Fig. 10, the interactions between oxide dispersed in SBA-15, pure silica matrix, and oxide of a mixture of chitosan and oxide are shown for the mechanistic model for methylene blue adsorption on various composite surfaces. However, the MB adsorption ability in the treated sample remained nearly consistent during the initial pH range of 5–11. (Fig. 11). One of the key factors determining the absorption of the methylene blue dye is the pH of the solution [66]. The pH 8 to pH 11 range was shown to be the best range for eliminating methylene blue. The high concentration of H^+^ ions in the solution, which creates competition with methylene blue, which is also positively charged [67], is responsible for the reduced absorption at low pH. Thus, pH 9 was employed to explore additional parameters once the methylene blue dye was removed. Fig. 12 depicts the Fe-γ–CS–SBA-15 adsorbent dose used to better bind methylene blue than Fe-SBA-15 for a 30-min period at pH 9. According to the findings of this experiment, 0.05 g of adsorbent dose was the right quantity for the adsorption of methylene.

#### 3.2.1 Adsorption isotherms

**Fig. 13:**
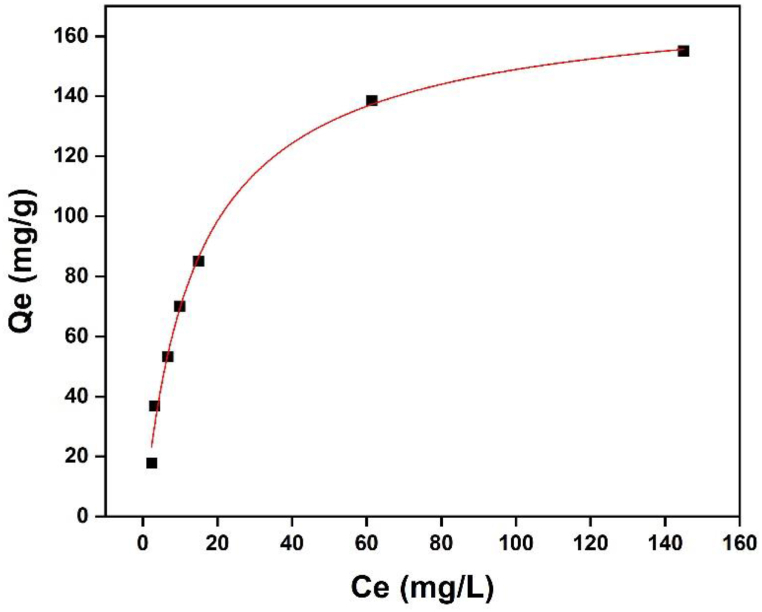
Adsorption isotherms of MB on Fe-γ–CS–SBA-15 (adsorbent dosage 0.05 g, [MB] 20–300 mg/L, Time 30 min, pH 9).

The interaction between the adsorbate molecules and adsorbent species in solutions was examined using Langmuir equations [[68], [69], [70]] to assess the adsorption data for methylene blue onto Fe-γ–CS–SBA-15. Isotherm model is represented as following equations:

(2) $$Qe=\frac{Q_{\max}K_{L}C_{e}}{1+K_{L}C_{e}}$$

where Ce (mg/L) is the equilibrium concentration of MB in solution, Q_e_ (mg/g) is the adsorbed amount of MB at equilibrium concentration, maximum adsorption capacity (Q_max_, mg/g) is the monolayer capacity of adsorbent, and K_L_ (L/mg) is the Langmuir binding constant. The Q_max_ value for adsorption isotherms is 176.70 mg/g. According to the findings reported in Fig. 13, the correlation value R^2^ calculated using the Langmuir isotherm adsorption model is 0.9945. The estimated Q_max_ values of the Langmuir equations are compared in Table 3 with results from earlier investigations [[71], [72], [73], [74]].

**Table 3:** Comparison of various adsorbents: adsorption capacity.

Adsorbent	Adsorbate	Q_max_	Refercence
Fe–Zn activated carbon	Methylene blue	169.779 mg/g	[71]
Carbon nanotubes/polyacrylonitrile	Methylene blue	172.41 mg/g	[72]
COK-12* Large-pore ordered mesoporous silica (OMS) COK-12	Methylene blue	20.2 mg/g	[73]
COK-12 grafted with GO *graphene oxide (GO)	Methylene blue	197.5 mg/g	
Mesoporous Iraqi red kaolin clay	Methylene blue	240.4 mg/g	[74]
Fe-γ–CS–SBA-15	Methylene blue	176.70 mg/g	This work

#### 3.2.2 Adsorption kinetics

**Fig. 14:**
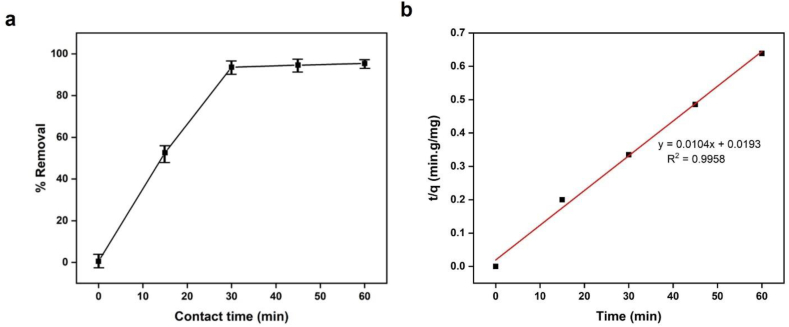
(a) Effect of contact time on the adsorption of methylene blue and (b) The pseudo-second-order adsorption kinetic equation (Fe-γ–CS–SBA-15 0.05 g, [MB] 100 mg/L, pH 9).

According to Fig. 14(a), methylene blue can be absorbed for up to 30 min before attaining an elimination effectiveness of 96%. Adsorption kinetics of methylene blue on Fe-γ–CS–SBA-15 was examined using pseudo-second-order model which gives a linear form as follows:

(3) $$\frac{t}{q_{t}}=\frac{1}{kq_{e}^{2}}+\frac{t}{q_{e}}$$

where k (g g^−1^ min^−1^) is the rate constant of pseudo-second-order adsorption; q_e_ and q_t_ (g mg^−1^) are the amounts of MB adsorbed at equilibrium and time t (min), respectively. Fig. 14(b) shows plot of the pseudo-second-order kinetic model for adsorption of MB on Fe-γ–CS–SBA-15. The pseudo-second-order model can adequately depict the adsorption of MB onto Fe-γ–CS–SBA-15 based on the coefficients R^2^ obtained from fitting, which is 0.9958 [71].

## 4 Conclusion

Fe-γ–CS–SBA-15 were successfully synthesized hydrothermally, and their characteristics were determined by XRD, HRTEM, EDS, FTIR, and N2-physisorption (BET, BJH). The best adsorption conditions were discovered to be pH 9 at room temperature, a contact time of 30 min, a maximum adsorption removal efficiency of 96% and the Q_max_ of the methylene blue is 176.70 mg/g. It is obvious that this mesoporous material has the potential to become a dye adsorbent with greater efficiency. The pseudo-second-order kinetics and Langmuir isotherm were both closely followed by the adsorption. The outcomes demonstrated that adding chitosan to the holder did not change its usual hexagonal structure, preserving its shape and a consistent distribution of pores.

## Author contribution statement

Titiya Meechai: Conceived and designed the experiments; Performed the experiments; Analyzed and interpreted the data; Contributed reagents, materials, analysis tools or data; Wrote the paper.

Thinnaphat Poonsawat, Artitaya Yatsomboon: Conceived and designed the experiments; Performed the experiments.

Nunticha Limchoowong: Conceived and designed the experiments; Contributed reagents, materials, analysis tools or data; Wrote the paper.

Sakchai Laksee, Ekasith Somsook: Contributed reagents, materials, analysis tools or data.

Peerapong Chumkaeo: Performed the experiments; Analyzed and interpreted the data.

Ranida Tuanudom: Conceived and designed the experiments; Wrote the paper.

Lalita Honghernsthit: Conceived and designed the experiments; Contributed reagents, materials, analysis tools or data.

Phitchan Sricharoen: Conceived and designed the experiments; Analyzed and interpreted the data; Contributed reagents, materials, analysis tools or data; Wrote the paper.

## Data availability statement

Data included in article/supp. Material/referenced in article.

## Declaration of competing interest

The authors declare that they have no known competing financial interests or personal relationships that could have appeared to influence the work reported in this paper
